# MetaProm: a neural network based meta-predictor for alternative human promoter prediction

**DOI:** 10.1186/1471-2164-8-374

**Published:** 2007-10-17

**Authors:** Junwen Wang, Lyle H Ungar, Hung Tseng, Sridhar Hannenhalli

**Affiliations:** 1 Center for Bioinformatics, University of Pennsylvania, Philadelphia, PA 19104, USA; 2 Department of Genetics, University of Pennsylvania, Philadelphia, PA 19104, USA; 3 Department of Computer and Information Science, University of Pennsylvania, Philadelphia, PA 19104, USA; 4 Core Genotyping Facility, Advanced Technology Program, SAIC-Frederick, Frederick, MD 21702, USA; 5 Division of Cancer Epidemiology and Genetics, NCI, NIH, Bethesda, MD 20892, USA; 6 Department of Dermatology, University of Pennsylvania, Philadelphia, PA 19104, USA; 7 Cell and Developmental Biology, University of Pennsylvania, Philadelphia, PA 19104, USA; 8 Center for Research on Reproduction and Women's Health, University of Pennsylvania, Philadelphia, PA 19104, USA

## Abstract

**Background:**

De novo eukaryotic promoter prediction is important for discovering novel genes and understanding gene regulation. In spite of the great advances made in the past decade, recent studies revealed that the overall performances of the current promoter prediction programs (PPPs) are still poor, and predictions made by individual PPPs do not overlap each other. Furthermore, most PPPs are trained and tested on the most-upstream promoters; their performances on alternative promoters have not been assessed.

**Results:**

In this paper, we evaluate the performances of current major promoter prediction programs (i.e., PSPA, FirstEF, McPromoter, DragonGSF, DragonPF, and FProm) using 42,536 distinct human gene promoters on a genome-wide scale, and with emphasis on alternative promoters. We describe an artificial neural network (ANN) based meta-predictor program that integrates predictions from the current PPPs and the predicted promoters' relation to CpG islands. Our specific analysis of recently discovered alternative promoters reveals that although only 41% of the 3' most promoters overlap a CpG island, 74% of 5' most promoters overlap a CpG island.

**Conclusion:**

Our assessment of six PPPs on 1.06 × 10^9 ^bps of human genome sequence reveals the specific strengths and weaknesses of individual PPPs. Our meta-predictor outperforms any individual PPP in sensitivity and specificity. Furthermore, we discovered that the 5' alternative promoters are more likely to be associated with a CpG island.

## Background

Initiation of transcription is regulated by a coordinated binding of many transcription factors to the core promoter region. The initiation process is further modulated by binding of activators and repressors in more distal regions [1,2]. The core promoter is the region (usually ± 50 bps) around the transcription start site (TSS), which is vital for initiation of the basal transcription. The core promoter contains several transcription factor binding sites that facilitate transcription initiation, such as the TATA box, the GC box, Inr [1,3], and the recently discovered MTE [4] and DPE [5]. In human, the TATA box is the most abundant, present in 25–30% of promoters within the entire genome [3,6]. The process of predicting the core promoter can therefore be summarized as using these characteristics to locate the TSS.

To understand eukaryotic transcriptional regulation, accurate identification and localization of core promoters are important [7]. The difficulty in identifying eukaryotic core promoters is that unlike in prokaryotes, eukaryotic promoters are sometimes located several hundred kb away from the translation initiation site (TIS). The eukaryotic promoters are usually identified by detecting full-length cDNA, e.g., oligo-capping [8]. However, such experimental methods are laborious, time-consuming and expensive. De novo computational Promoter Prediction Programs (PPPs) show great potential in this regard and have achieved moderate success in the past [9,10]. Nevertheless, promoter prediction at high resolution, especially for promoters that are not associated with CpG islands (CpG-poor promoters), remains unsatisfactory [11-14].

It is widely recognized that promoter regions are correlated with CpG islands. CpG islands are regions of DNA longer than 200 bps with a G+C content of at least 50%, and the number of CpG dinucleotides being at least 60% of what could be expected from the G+C content [15,16]. CpG islands are well known to be highly associated with many mammalian gene promoters (CpG-rich promoter); about 50~60% of the promoters are associated with CpG islands [17]. The first generation PPPs, such NNPP [18], TSSG and TSSW [19], PromFD [20], and PROSCAN [21] did not use CpG island as a landmark and thus showed poor results in large-scale evaluations [2]. A consensus program, CONPRO [22], combined features of these PPPs and other genomic information for promoter prediction. Tested on a small dataset, CONPRO showed improvements over individual PPPs. Various other techniques were also employed with varying success. Homology-based promoter predictions have achieved moderate success [23,12]. PromoterInspector [24] improved prediction accuracy by allowing variable gaps between fixed oligomers, and implicitly using CpG island information [11]. Zhang's group [25] was the first to classify explicitly the promoters into CpG-island-associated and the non-CpG-island-associated. They implemented this notion (i.e., using CpG island as landmark) in their recent promoter prediction program – FirstEF, and achieved significant improvement [9]. Since then, all the high performing programs, such as DragonGSF [26], McPromoter [27], and PSPA [13] use CpG islands as landmarks to make a prediction.

To evaluate fairly the performance of the PPPs, we separated promoters into two subtypes – CpG-rich and CpG-poor, depending on whether they are CpG-island related or not [28]. To ensure that a CpG-poor promoter does not relate to any CpG island, we classified each promoter as follows. If a CpG island is present within the ± 5 kbps sequence of a promoter, we classified the it as CpG-rich, otherwise CpG-poor [13,28]. This classification adopts a more stringent criterion for a TSS being non-CpG-related (CpG-poor). Thus, in our study, the proportion of CpG-rich promoters is higher than the previous estimates [12].

Different programs utilize different characteristics of the genomic sequence near the promoter to make predictions. For example, DragonGSF and DragonPF use CpG islands as a global landmark and integrate additional attributes using an Artificial Neural Networks (ANN) to predict TSSs within the ± 3700 bps of CpG islands [26,29]. FirstEF first scans -1,500 to +500 bps to detect a CpG island, then uses two different quadratic discriminant functions in a -500 to +70 bps window for TSS prediction [9]. These attributes include the frequencies of fixed-length motifs in different windows in the -500 to +70 bps region. The FProm program uses a linear discriminant function to make prediction based on the characteristics in the -200 to +50 bps region of the TSS [19,30]. McPromoter focuses on the -250 to +50 bps region and uses a generalized hidden Markov model, with six interpolated Markov chain submodels representing different segments of the promoter region [27]. The recently improved version of McPromoter classifies drosophila promoters into 5 subtypes and uses one model for each subtype to make a prediction [14]. PSPA uses -100 to +100 bps around the TSS and uses a strict position-specific and variable-length motif propensity model. It shows a superior performance on CpG-poor promoters [13]. Based on a large-scale evaluation on the human genome, a recent review [10] showed that DragonGSF and FirstEF performed better at a low resolution (i.e., cutoff at 2000 bps). Another recent evaluation [13] showed that their performances deteriorated sharply at a more stringent resolution (cutoff <500 bps). The study showed that DragonGSF made virtually no predictions on the CpG-poor promoters and FirstEF made no prediction on 85% of the CpG-poor promoters. Even though PSPA improved prediction on the CpG-poor promoters, its overall performances on CpG-poor promoters remained unsatisfactory. A focus on high resolution and on CpG-poor promoter prediction is needed [10,13].

The previous evaluations [10] were limited to the most upstream TSS (MUTSS) or the most frequent TSS as defined by DBTSS database [8]. Recent studies showed that there were several hundred thousand TSS in the human and mouse genomes, and 58% of the mammalian genes have alternative transcription start sites (ATSS) [31]. The presence of multiple ATSS for a single gene is related to tissue-specific gene expression. For example, the UDP-glucuronyl transferase gene has seven alternative promoters, each responsible for expression in a certain type of tissue [31]. The more recent ENCODE region study showed that the regulatory elements distributed symmetrically around the TSS, with no bias towards the upstream regions [32]. As more and more experimentally validated ATSSs become available, it is necessary to evaluate the PPPs on all TSSs (termed ATSS, alternative TSS in this paper), not only the MUTSSs. Furthermore, promoters predicted by various PPPs do not overlap. At a prediction resolution of -50 to +50 bps, the correct predictions from different programs are largely distinct [13]. If a meta-predictor could combine the correct predictions from each PPP, it would achieve a much higher performance.

**Figure 1:**
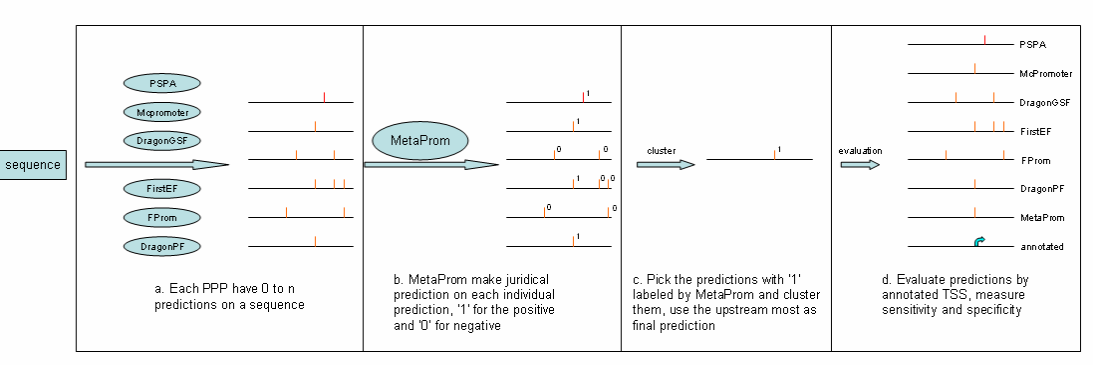
**Flowchart illustrating MetaProm prediction and evaluation**. For each of the 14,599 sequences, **A**) six PPPs were used to make predictions. The predictions, as well as the genomic context information, were extracted and compiled to 28 features. **B**) The ANN-based MetaProm is trained and tested on these features, the program either accepts or rejects a prediction. **C**) The program pools all accepted predictions and clusters them and takes the most upstream one as meta predictions. **D**) The meta predictions, along with predictions from PPPs, were evaluated.

In this paper, we first describe the relationship between CpG island and promoters. We then evaluate the performance of PPPs on a large set of ATSS, which includes MUTSS as well as the other promoters, including the middle TSS (MTSS) and the most downstream TSS (MDTSS). Finally, we introduce a meta predictor that combines promoter predictions from top-performing PPPs using Artificial Neural Networks, as well as the genomic information such as CpG island (Figure 1). Our large-scale tests on the human genome show that the meta predictor is significantly superior in terms of sensitivity and specificity, as compared to the individual PPPs.

## Results

### Alternative promoters are symmetrically distributed

**Figure 2:**
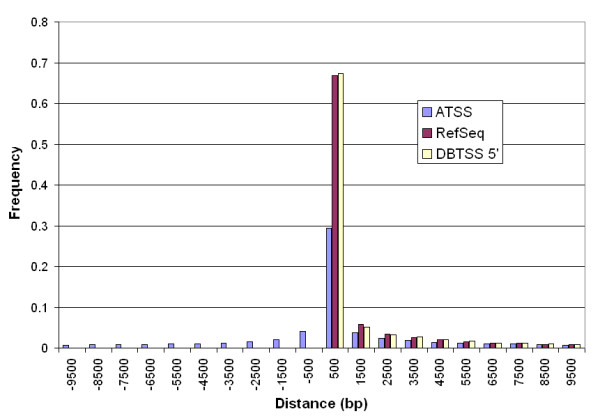
**Histogram of distances between Transcription Start Site (TSS) and Coding Start (CDS)**. ATSS: based on 30,964 Alternative TSS from DBTSS database; RefSeq: based on 25,647 TSS from RefSeq database; DBTSS 5': based on 14,628 most upstream TSS from DBTSS database, a subset of ATSS. All data are binned by size of 1 kb, with registered on the x-axis by the middle point. Positive values in the x-axis indicate TSS is upstream of CDS. Note that there is no significant difference between RefSeq and DBTSS 5'. ATSS from DBTSS is present both up- and down-stream of CDS, with a symmetrical distribution around the bin of 500.

We used TSS annotations from DBTSS[33] and RefSeq [34] as our reference. Since DBTSS includes alternative TSS, we extracted the must upstream TSS as a subset, named DBTSS 5'. We compared the distance between MUTSS and the upstream coding sequence (CDS) documented in these databases and found no significant difference (Figure 2). Both DBTSS 5' and RefSeq annotated TSSs were upstream of the CDS, and about 67% were within 1 kb upstream. However, when DBTSS ATSSs were counted, only 30% of the ATSSs were within 1 kb upstream of CDS, and the rest distributed symmetrically around this region.

We then integrated the TSSs from the two datasets into one and clustered them based on their genomic locations. If twoTSSs are less than 5 bps apart, we take the upstream one as a representation of the cluster. After removing the redundant TSSs, we obtained 42,536 distinct ATSS. We pooled these ATSS together and clustered them into 14,566 clusters (see Methods). For each cluster, we extracted the region spanning 5 kb upstream of the MUTSS to the end of the gene (if there were multiple genes in the cluster, we used the MUTSS of the first gene and 3' UTR of the last gene). We thus obtained 14,566 sequences, with a total of 1.06 × 10^9 ^bps, which equaled approximately 30% of human genome. These sequences were used for promoter prediction. Among the 42,536 ATSS, there are 14,566 MUTSS, 13,114 MDTSS, and 14,856 MTSS. The distance distribution among these TSSs is shown in Additional file 1.

### Upstream promoters are more frequently associated with CpG islands

**Figure 3:**
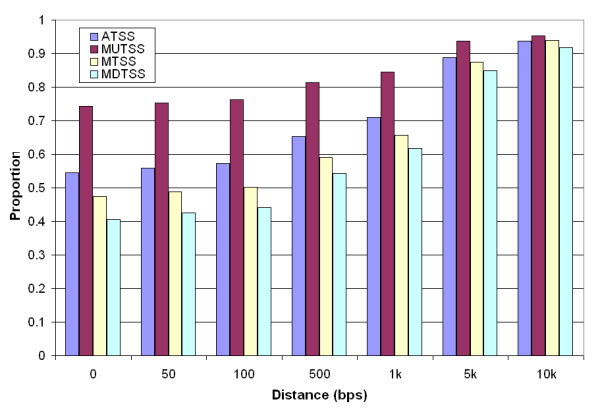
**Distance between Transcription Start Site (TSS) and CpG island**. ATSS: all promoters; MUTSS: most upstream promoter (most 5' promoter); MTSS: middle promoter; MDTSS: most downstream promoter. Zero distance indicates the TSS is within the CpG island. The number in longer distance includes that in the short distance. MUTSS has 80% more chance to be in CpG island than MDTSS.

Since CpG islands play a vital role in promoter prediction, we analyzed the correlation between CpG islands and human gene promoters. To illustrate the relationship between different types of promoters, we classified the promoters as MUTSS, MDTSS and middle promoters (MTSS), depending on their locations in the cluster. We used a less stringent CpG island detection program [35], which were used in our previous study [13]. Among the 14,566 sequence, the program detected 162,726 CpG islands. Figure 3 shows the distance between CpG islands and the three types of ATSS. 74% of the MUTSS were within a CpG island, approximately 81% were within 500 bps, and about 95% were within 10 kbps of a CpG island. The numbers for MDTSS were substantially lower, only 41% were within a CpG island and 54% within 500 bps of a CpG island. The corresponding numbers for all promoters were 55%, 65%, and 94% respectively.

We also used the CpG islands that were annotated in UCSC genome browser [36] and observed the same pattern (Additional file 2). The database included 20,238 CpG islands in our promoter sequences. Of the MUTSS, 60% were within a CpG island, approximately 70% were within 500 bps, and about 78% were within 10 kbps of a CpG island. In contrast, of the MDTSS, only 31% were in CpG islands, 37% were within 500 bps, and 54% within 10 kbps.

### Statistics of promoter prediction by individual PPP

The 14,566 sequences were used for promoter prediction by each PPP. We obtained a total of 339,960 TTS-predictions from six PPPs. We then classified the predictions into two categories. If a prediction was within ± 5 kb of a CpG island, it was classified as a CpG-rich prediction otherwise a CpG-poor prediction. The classification resulted in 247,540 (72.8%) CpG-rich predictions and 92,420 (27.2%) CpG-poor predictions. The composition of the predictions contributed by each PPP was shown in Table 1. FProm made the majority of the predictions, 77% of the total CpG-rich predictions and 92% of the CpG-poor predictions. Each of the other PPPs made about 5% of the total CpG-rich predictions. Three PPPs, DragonGSF, FirstEF and McPromoter made few CpG-poor predictions.

**Table 1:** The proportion of predictions made by each promoter prediction program.

**PPP**	**CpG-rich**	**CpG-poor**
**DragonGSF**	4.13%	0.00%
**DragonPF**	3.91%	0.58%
**FirstEF**	4.42%	0.00%
**FProm**	76.58%	92.29%
**McProm**	4.83%	0.09%
**PSPA**	6.13%	7.04%

With total 339,960 predictions in 30% of human genome by six PPPs, 247,540 (72.8%) are CpG-rich predictions and 92,420 (27.2%) are CpG-poor predictions.

We then looked at the average number of predictions made by each PPP per promoter. As shown in Table 2, FProm was very lenient in making predictions; it made on average five predictions for each CpG-rich promoter and 18 predictions for each CpG-poor promoter. The other five programs were comparable on CpG-rich promoters. On average, they make about 0.25 to 0.4 predictions per true CpG-rich promoter. On CpG-poor promoters, PSPA made about 1.4 predictions per true CpG-poor promoter, and DragonPF made about 0.11 predictions.

**Table 2:** Average predictions per true promoter by each promoter prediction program.

	**DragonGSF**	**DragonPF**	**FirstEF**	**FProm**	**McProm**	**PSPA**
**CpG-rich**	0.27	0.26	0.29	5.02	0.32	0.40
**CpG-poor**	0.00	0.11	0.00	17.96	0.02	1.37

### Performance evaluation of individual PPP

**Figure 4:**
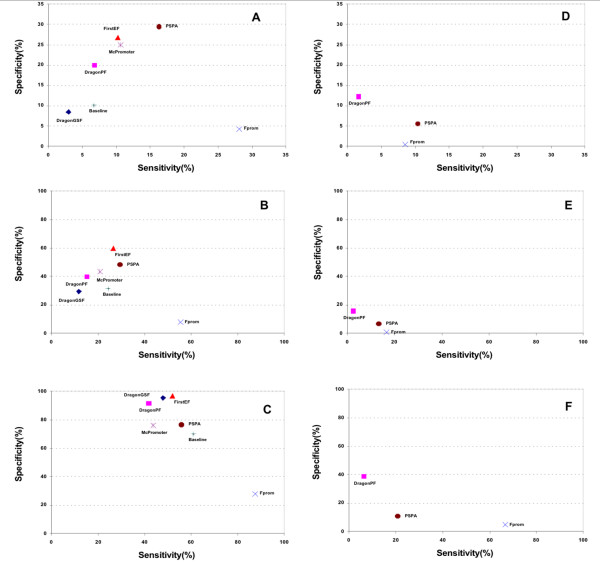
**Performance of PPPs on genome-wide prediction**. **A**, **B**, **C**: PPPs' performances on CpG-rich sequence at **A**) high (50 bp), **B**) medium (200 bp) and **C**) low (2 kbp) resolution, respectively. **D**, **E**, **F**: Performances on CpG-poor sequence at **D**) high (50 bp), **E**) medium (200 bp) and **F**) low (2 kbp) resolution, respectively. The performance is measured by sensitivity, which is the proportion of all true promoters that are predicted correctly, and specificity, which is the proportion of total prediction that are correct. The evaluation was based on 37,793 CpG-rich promoters and 4,743 CpG-poor promoters on 1.06 × 10^9 ^bps of the human genome. For baseline prediction, we repeated the randomization (see text) 10 times and the means are reported. The standard deviations (sensitivity, specificity) are (± 0.2%, ± 0.3%), (± 0.2%, ± 0.2%), (± 0.0%, ± 0.1%) for the high, medium and low resolutions, respectively.

We evaluated the performance of six PPPs on CpG-rich and CpG-poor promoters separately. The sensitivity and specificity of each PPP were reported at three levels of resolution – high (50 bps), intermediate (200 bps) and low (2000 bps). All the predictions were subject to the same evaluation criteria and the results were shown in Figure 4A–F.

For CpG-rich promoter evaluations, we added a baseline prediction as a control. To perform the baseline prediction, we picked a random location near each CpG island (defined as such in the UCSC genome browser) [37], and used it as a prediction. A total 20,238 random predictions were evaluated by the same criteria as other PPPs, and sensitivity, specificity were reported. To evaluate the variation of the random baseline prediction, we repeated the process 10 times. The standard deviations for sensitivities and specificities were also reported in the figure legend. Figure 4A–C showed the performance of six PPPs and the baseline prediction on CpG-rich promoters. PSPA and FirstEF performed better at high resolution, whereas FirstEF and DragonGSF performed better at low resolution. FirstEF, PSPA and McPromoter performed better at intermediate resolution. FProm had a higher sensitivity, but a lower specificity at all resolutions. FirstEF and PSPA both performed relatively well with a balanced sensitivity and specificity. The baseline prediction, though randomly selected within a CpG island, performed very consistently, with standard deviation of only ± 0.0% to ± 0.3% for both sensitivity and specificity.

We evaluated the performance of DragonPF, FProm and PSPA on CpG-poor promoters (Figure 4D–F). We excluded other PPPs from this evaluation because they virtually did not make predictions on the CpG-poor promoters. In general, the performance on CpG-poor promoter was much lower than that of the CpG-rich promoter. At a low resolution, DragonPF showed a higher specificity and FProm a higher sensitivity. PSPA showed a higher sensitivity at high resolution and had a balanced sensitivity and specificity in all ranges of resolutions.

To evaluate the similarity of these PPPs, we compared the correct predictions from each pair of PPPs at medium resolution (200 bp) and the results were shown in Tables 3 and 4. The overlaps in CpG-rich promoter (Table 3) were substantially larger than that of the CpG-poor (Table 4). For promoter predictions in CpG-rich sequences, FProm and DragonPF had the highest overlap at 81%, and McPromoter and DragonGSF had the lowest at only 38%. For promoter prediction in CpG-poor sequence, FProm and PSPA had the highest overlap at 26%, and FirstEf and FProm had the lowest overlap at 12%. The overlaps at high and low resolutions were shown in Additional file 3. With less restricted resolutions, we observed a sharp increase of overlaps from the high to low resolutions for CpG-rich sequences. The overlaps are 11%~52% for the high resolution, 38%~81% for the medium resolution, and 66%~89% for the low resolution. However, the trend was not as significant for the CpG-poor sequences, where predictions overlapped at 6%~32%, 12%~31%, and 18%~35% for the high, medium and low resolutions, respectively.

**Table 3:** Pair-wise overlaps of correct predicted promoters between two PPPs at medium (200 bp) resolution for the CpG-rich promoters.

	**PPP (correct predictions)**					
	
		**DragonGSF **(7,205)	**DragonPF **(18,959)	**FirstEF **(15,015)	**FProm **(20,884)	**McProm **(7,891)
**PPP (correct predictions)**	**DragonPF **(18,959)	49% (6,380)				
	**FirstEF **(15,015)	44% (4,890)	75% (12,795)			
	**FProm **(20,884)	45% (6,310)	81% (16,084)	70% (12,632)		
	**McProm **(7,891)	38% (2,870)	52% (6,918)	49% (5,636)	49% (7,051)	
	**PSPA **(11,164)	47% (4,313)	67% (10,091)	67% (8,750)	61% (9,806)	48% (4,534)

The number in parenthesis is the count of correctly predicted promoters; the percentage number is calculated by the count of the overlap divided by the mean of both PPPs. The overlaps at high and low resolutions are shown in Additional file 3.

**Table 4:** Pair-wise overlaps of correct predicted promoters between two PPPs at medium (200 bp) resolution for the CpG-poor promoters.

	**PPP (correct predictions)**			
	
		**DragonPF **(199)	**FirstEF **(183)	**FProm **(789)
**PPP (correct predictions)**	**FirstEF **(183)	15% (28)		
	**FProm **(789)	27% (134)	12% (56)	
	**PSPA **(629)	19% (80)	26% (104)	31% (223)

### CpG islands is key to prediction accuracy

Another notable feature in Figure 4 was that the baseline prediction, particularly at the low resolution, performed as well as the other PPPs. This result showed the power of using CpG-islands as a landmark for promoter prediction, and how current PPPs relied on CpG island critically. Even though they would miss the promoters that were not associated with CpG islands, PPPs that made prediction exclusively on regions near CpG islands were bound to perform better than those PPPs that did not use CpG islands as a landmark.

The promoter prediction problem was much harder for CpG-poor sequences. Since there was no CpG island to serve as the landmark, the PPPs had to consider a much larger region for prediction. An alternative approach was to use the feature of a gene as a landmark, since gene prediction programs use context information derived from a higher degree of conservation in the encoding region. It was shown that integration of gene prediction and EST information improved promoter prediction [22,38]. A recent study [13] showed that CpG-poor promoters were more conserved and had fewer alternative start sites than the CpG-rich promoters. This observation was further confirmed [31,39]. The observation implied that the signal around CpG-poor promoters was stronger, and was independent of CpG islands. The promoter prediction on CpG-poor promoters was thus likely to be more accurate. However, most state-of-the-art PPPs used CpG islands as a primary landmark, which reduced the search scope by about 50-fold. As a result, prediction accuracy on CpG-rich promoters was far higher than that on the CpG-poor promoters.

### Evaluation of MetaProm

**Figure 5:**
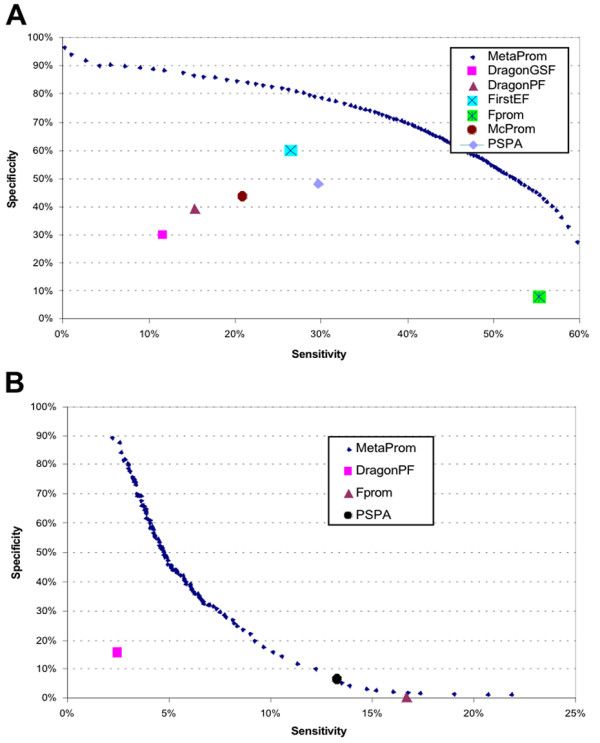
**Evaluation of MetaProm at medium resolution on genome-wide promoter prediction**. **A**) On CpG-rich promoters; **B**) On CpG-poor promoters. The evaluations of MetaProm are based on the 10 cross-validation. The evaluations on the high and middle resolutions are shown in Additional file 4.

Next we used a neural-network-based approach to integrate the predictions made by individual PPPs to improve the overall prediction accuracy. We included the performance of the different PPPs at the intermediate resolution of 200 bps. The performance of MetaProm was based on 10-fold cross-validation. The sensitivity~specificity coordinate of each PPP was shown in Figure 5. MetaProm was effective on CpG-rich promoter prediction. At a specificity of 60%, MetaProm boosted the sensitivity from 26% to 46% as compared to FirstEF. At a specificity of 50%, it improved the sensitivity from 29% to 52% as compared to PSPA. On CpG-poor promoter predictions, MetaProm made only marginal improvements upon the current best performer, PSPA [13]. This was because only three programs make predictions on CpG-poor promoters, and they made very few predictions. The evaluations of MetaProm at the high and low resolutions were shown in Additional file 4.

## Discussion

It is widely recognized that promoter regions are correlated with CpG islands. CpG islands were originally found around TSSs in about 55% of the human promoters, based on hundreds of experimental screening of human genes [40]. Since then, CpG islands have been used as landmarks in many promoter prediction programs [9,29,27,13]. Recent large-scale oligo-capping of full-length cDNA techniques provided a large set of experimentally validated promoter data, which enable us to evaluate the links between CpG islands and promoters in much greater detail [31]. Using an older version of RefSeq, previous study showed that 34% of the RefSeq annotated TSSs could be extended towards the 5' ends [8]. Here we used a recent version of RefSeq database [41], which was substantially enhanced and includes most of the TSS from DBTSS. Our study showed that there was not a significant difference between the RefSeq and DBTSS MUTSS annotations in terms of distance to the translation start site.

Since the discovery of the close association between CpG islands and promoters, this association has been widely utilized for promoter and gene prediction. Previous studies showed that about 50~60% of the promoters are associated with CpG islands[17], we found the association is stronger. By using a recently developed CpG island calculation program [35], we showed that more than 65% of all promoters are within 500 bps of a CpG island, and 95% of all promoters are within 10 kbps of a CpG island.

More importantly, we found that the 5' alternative promoters were more closely linked to CpG islands than the 3' promoters of the same gene. Consistent with the role of CpG islands in the recruitment of chromatin modification enzymes, it is conceivable that the most upstream promoters represent the broadly used substrate in a hierarchical regulation of gene transcription, whereas the downstream, non-CpG-associated promoters are used in a tissue-specific fashion in conjunction with the upstream promoters. We hypothesize that most polymerase II transcription complexes are assembled at the vicinity of a CpG island. It then either starts transcription, or slides to another active promoter to initiate transcription.

Our results also showed that improvements on CpG island prediction can further reveal the relationship between CpG islands and promoters. Since the new CpG island program detects substantially more CpG islands, not surprisingly, we found more promoters are associated with CpG islands. It also provides us a challenge to develop CpG island detection programs that can help identify promoters. We believe this is especially important in the current context of DNA methylation and histone modification studies [42,43]. The CpG island detection program will give us more opportunity to understand not only genetics, but also the epigenetic regulation of genes. Similar to the discovery of the higher proportion of promoters associated with CpG islands, we might find that more DNA methylation and histone modification events are associated with CpG islands.

Different PPPs capture different characteristics of mammalian core promoters. Because most PPPs are based on machine learning approaches, the genomic attributes captured by the PPPs are not thoroughly investigated. These attributes will be important in understanding mammalian promoters and in return help us to develop a better PPP. As a first step, we propose a MetaProm tool that integrates the predictions by individual PPPs using an artificial neural network. By combining these predictions, our MetaProm showed significant improvement over the individual PPPs. Liu and States [22] have developed a consensus method -CONPRO. The authors were able to improve the prediction accuracy by combining results from five PPPs and one gene prediction program. However, the five PPPs (NNPP [18], TSSG and TSSW [19], PROSCAN [21] and PromFD [20]) used were shown to perform poorly [44]. In contrast, the PPPs used in our meta-predictor are relatively new and all are different from the ones CONPRO used, except FProm, which is an improved version of TSSG.

Several reasons contribute to MetaProm's lack of improvement on CpG-poor promoter prediction. First, only three PPPs make predictions on this type of promoters and the number of predictions is much lower comparing to the CpG-rich promoters. Second, the overlaps between the three PPPs are also substantially lower than that of CpG-rich promoters. Third, the proportion of overlap does not increase as we go from a high resolution to a low resolution (Table 4). Since MetaProm does not make new predictions, it relies on the context information from other PPPs in the surrounding region. For CpG-rich promoters, the overlaps are as high as 80% in medium and low resolution, and the model can use context information from other predictions and thus works better. However, for CpG-poor promoters, 1) we do not have sufficient predictions to use and 2) these predictions usually do not overlap in the 2 kb base pair region, which is the maximum context information that our model uses. Therefore, the improvement is not significant.

The recent large-scale determination of full length cDNAs has generated large amount of reliable promoter data, and has led to some novel insights. For example, recent data shows that 58% of genes have multiple alternative start sites and these often correspond to tissue-specific expression of the transcript [31]. In this study, we have included alternative TSS to evaluate the current PPPs for the first time. Not surprisingly, as more and more annotated TSSs are considered, we get higher specificity and lower sensitivity. We are aware that most PPPs were trained and tested only for MUTSS prediction, which may underestimate their performances. However, since the biological paradigm is shifting from one gene, one promoter to one gene, multiple promoters, it also imposes computational challenge to the promoter prediction field.

Even though the large-scale experimental data provide us with a large number of cDNAs, these cDNAs are by no means comprehensive and exhaustive. Some false positive predictions by the MetaProm program might prove to be true positives once the experimental detection of promoters becomes more sensitive. The core promoter prediction programs also provide a basis for designing the whole-genome promoter array. Furthermore, algorithms that are successful on human promoter prediction can hopefully be used in other mammalian genome promoter prediction, and thus guide experimental studies.

Previous evaluation on MUTSS reports greater variability of the performance on different chromosomes [10]. Our evaluation on ATSS shows that most PPPs have consistent performance on different chromosomes (data not shown). Every PPP seemingly captures slightly different attributes of the promoter sequences and thus makes predictions that are largely unique to the PPP [13]. A recent paper discussed extensively the attributes used by each of these PPPs [38]. DragonPF, DragonGSF and firstEF incorporate both promoter region and part of gene structure to make a prediction, and thus require a relatively longer sequence (>500 bps). FProm, McPromoter and PSPA use shorter sequences (<250 bps) flanking the promoter region to make predictions. They do not depend on protein-coding region thus can be used in predicting RNA gene promoters. Until we gain a better understanding of the biological signals encoded in the promoter regions that are recognized by the transcriptional machinery, our machine-learning approach to integrate the predictions made by different PPPs will provide a valuable resource.

While this manuscript was under review, another promoter prediction program [45] was published, with focus on non-CpG-related promoter prediction. The program adopted a LogitBoost procedure to make prediction based on features such as position-specific elements, TFBS, and k-mer frequencies. This study differs from our study in the following: 1) Similar to other state-of-the-art PPPs, this study focuses on MUTSS prediction, whereas our study focuses on ATSS prediction; 2) the assessment of the program is performed on a limited length of sequence (2.4 kb around the annotated TSS). We focus on the whole genome prediction, with about 30% of the human genome sequences; 3) the assessment is based on a smaller dataset (trained and tested on 3,210 CpG-related promoters and 1,576 non-CpG-related promoters). Our study uses 37,793 CpG-rich promoters and 4,743 CpG-poor promoters. This new program gains significant improvement on both CpG-related and non-CpG-related promoter predictions. Incorporating this PPP into our meta predictor has the potential to improve overall prediction performance.

## Conclusion

Our genome wide evaluation was based on all available promoters, including alternative promoters. We discovered that promoters at the 5' end of the gene are more likely to be linked to a CpG island. Evaluation based on the human genome shows that MetaProm performs better than any of the individual PPP both in terms of sensitivity and specificity. This meta prediction method should be useful in locating the promoter region of a gene, and thus facilitating the analysis and understanding of gene regulation. The MetaProm program and the genome wide predictions are available upon request.

## Methods

### Dataset

We retrieved the coordinates of full-length cDNA sequences from the DBTSS [8] and RefSeq [34] databases, and mapped them to the human genome (version hg17). The sequences in DBTSS are comprised of full-length cDNA transcripts, whose 5' ends are experimentally determined [33]. The sequences in RefSeq are primarily from the GenBank repository and are manually-curated full-length cDNAs [41]. These two datasets are standards in genomic annotation and promoter analysis. In the latest version of DBTSS "near-by" genes are clusters into groups [33]. Each cluster contains one or more alternative TSS, and many groups contain multiple transcripts or genes (a transcript is defined by a unique 'NM' id). For each NM id, we also find the TSS annotated by RefSeq, and treat them as additional ATSS if they are more than 5 base pairs apart from the DBTSS annotation. The final dataset contains 14,566 groups (clusters) with 42,536 distinct ATSS. For each group, we extracted the genomic sequence that spans from 5 kbs upstream of the MUTSS to the end of the gene (end of 3' UTR). The sequence length varies from 5,243 bps to more than 2 million bps, with a combined sequence length of 1.06 × 10^9 ^bps, or about 30% of the human genome. Among these sequences, only 326 (2.2%) sequences do not contain any CpG island.

Out of 42,536 total ATSS, we have 37,793 (88.9%) CpG-rich promoters and 4,743 (11.1%) CpG-poor promoters; this proportion is similar to the that of MUTSS [13]. CpG islands are defined as GC enriched sequences of >200 bps, G+C content >50% and CG di-nucleotide ratio >0.6. We used a CpG island detection program based on [35], which adopted new criteria for CpG islands; these criteria result in more genes being associated with CpG islands. We separated the promoters into three groups based on their position in the cluster, the MUTSS, the MDTSS, and if there are more than two promoters in the cluster, we name them MTSS. All the promoters are called alternative promoters (ATSS). For the promoter-CpG island association study, we also downloaded the CpG island data from UCSC genome browser [37], which adopted a traditional definition of CpG islands and includes fewer CpG islands.

### Promoter predictions and parameter selection

Promoters are predicted on each sequence by individual PPP with default parameter settings as described in [10,13]. Briefly, the parameter settings for individual PPP are as following:

DragonGSF: threshold 0.994 (default)

DragonPF: sensitivity 0.65

FirstEF: default setting of P(Exon)>0.5, P(Donor)>0.4, and P(Promoter)>0.4.

McPromoter: threshold = -0.05

FProm: default setting

PSPA: score cutoff = 100 for CpG-rich, and cutoff = 150 for CpG-poor.

Similar to the classification for real promoters, we classified each predicted TSS into CpG-rich or CpG-poor based on whether there is a CpG in the surrounding ± 5 kbps region. The performances of individual PPPs are evaluated separately on CpG-rich and CpG-poor predictions. We also added a baseline prediction as control for CpG-rich promoter prediction. In each of the annotated CpG island [37] in the 14,566 sequences, we randomly choose a location within the CpG island as a prediction. The baseline predictions were subjected to same evaluation as other PPPs. We repeated the baseline prediction 10 times. The mean and standard deviation of the sensitivity and specificity were also reported.

### Evaluation of the predictions

We adopted the evaluation approach as previously described [10]. Because the promoter annotation is not complete, we do not know if the predictions in intergenic region are false positives, we only evaluate the predictions that fall within 2 kbs upstream of the MUTSS and the end of the gene (we call them valid predictions). A prediction is considered correct if it is ± L bps away from any of the annotated ATSS. We focus on three values of L in this study, 2000 bps to test the performance at low resolution, 200 bps to test at intermediate resolution, and 50 bps to test the performance at high resolution. The performances of PPPs are evaluated based on sensitivity and specificity. Sensitivity = (correctly predicted promoters)/(total number of promoters, including all ATSSs), Specificity (ppv) = (correct predictions)/(valid predictions). Since there can be more than one ATSS in a sequence, here a promoter is defined for an individual ATSS, not for a sequence (group or cluster). The pair-wise overlap of predictions by two PPPs, A and B, is calculated by O_AB _= (C_AB _× 2)/(C_A _+ C_B_), where is the number of correct predicted ATSS by both A and B, C_A_, C_B _are the numbers of correctly predicted ATSSs by A and by B, respectively.

### Features extracted for MetaProm

For each prediction, the MetaProm program makes a decision on how reliable the prediction is. The decision is based on the features we extracted from the genomic context, the prediction itself and the other two closest predictions in the surrounding region, either by the current PPP or by other PPPs. We observed that for the CpG-rich promoters, the overlap of correct predictions between PPPs increased rapidly from 50 bp resolution to 200 bp resolution and further to 2 kb resolution (Table 3 and Additional file 3). This implies that even though there is a small chance that two PPPs will predict the same location as a promoter, there is a greater chance that the different PPPs will make a prediction in the relative vicinity. Integrating other genomic information, such as the presence of a CpG island, the GC content, the CpG dinucleotide content and the length and location of the CpG island, allows the Artificial- Neural-Net-based model to make a better prediction.

We classified all predictions into 247,540 (72.8%) CpG-rich and 92,420 (27.2%) CpG-poor predictions. The two groups were trained and tested separately. For each prediction from any PPP, a total of 28 features were extracted from the individual prediction and its surrounding predictions. The MetaProm used these features to calculate the likelihood of this prediction to be true (Figure 1). The detailed descriptions of the features are given in Additional file 5. These features fall in three classes:

Features of the current prediction: which PPP made the prediction, the prediction score, is the prediction CpG-rich or CpG-poor. For some PPPs that do not provide prediction scores, we use the rank value.

Statistics on neighboring predictions: for example, how many predictions are made by other PPPs within a certain distance (50, 100, 500, 1 k, 2 k bps) away from current prediction. We also used the attributes of the closest prediction by any of other PPPs, such as which PPP makes the prediction, whether the prediction is CpG-rich or CpG-poor, the prediction score, and distance from current prediction. We also use the same attributes of the second closest prediction.

Finally, we used the attributes of the closest CpG island (or no CpG island for CpG-poor predictions). These include the length of the CpG island, G+C content, GC observed expected ratio, whether the prediction is in the CpG island, or 100, 200, 500, 1 k, 2 k, 5 k bps away from the edge of the closest CpG island and the distance of the prediction from closer side of the CpG island, farther side, and central of the CpG island.

### The MetaProm prediction

The MetaProm does not make new predictions, it recalculates score (probability) of each predicted promoter of being real. Every prediction from each individual PPP, along with their features, was used as one instance to the MetaProm Artificial Neural Network (ANN) model for training and testing. We used the MultilayerPerceptron function in the Weka package [46] to perform a 10-fold cross-validation using a back-propagation algorithm. We used a three layered structure (input, hidden and output layer). The input layer had 28 nodes (corresponding to 28 features as shown in Additional file 5) and the hidden layer had 15 nodes. The following parameter settings were used for both training and testing; learning rate 0.3, momentum rate 0.2, number of epochs 500, number of nodes in hidden layer is (input nodes + labels)/2.

The prediction accuracy for the MetaProm is obtained by the 10-fold cross-validation: the dataset is partitioned into 10 equal parts, and the ANN model is iteratively trained on nine parts and tested on the remaining part. The predictions with an ANN score above the cutoff (selected based on ROC curve) were taken as positives, and were clustered if they were within 5 bps with each other. The prediction with the highest ANN score in the cluster was selected as the final prediction. To draw the sensitivity-specificity curves of the MetaProm prediction, we pooled predictions from the 10-fold cross-validation and ranked the MetaProm prediction scores (probabilities), and selected different cutoffs to get the Sensitivity-Specificity pairs at that cutoff. Since we were not able to obtain the training versions of PPPs other than our own PSPA, we could only obtain one Sensitivity-Specificity pair for each predictor, where the cutoffs were pre-determined by the individual developer.

## Competing interests

The author(s) declares that there are no competing interests.

## Authors' contributions

JW conceived, designed and coordinated the study, implemented the software, performed the analysis and drafted the paper.

LHU contributed to the design of the study, and manuscript editing.

HT contributed to discussion and manuscript editing.

SH contributed to the design of the study, and manuscript editing.

All authors have read and approved the final manuscript.

## Supplementary Material

Additional file 1 Distances between alternative TSSs within a sequence. Click here for file

Additional file 2 Distance between Transcription Start Site (TSS) and CpG island (annotated in UCSC). Click here for file

Additional file 3 Pairwise overlaps of correct predicted promoters between each PPP at high (50 bp) and low (2 kb) resolutions. Click here for file

Additional file 4 Evaluation of MetaProm at high and low resolutions on genomewide promoter prediction. Click here for file

Additional file 5 Features used for ANN based MetaProm promoter prediction. Click here for file
