# Supplementary material for: MetaProm: a neural network based meta-predictor for alternative human promoter prediction
Source: BMC Genomics. 2007 Oct 17;8:374. doi: 10.1186/1471-2164-8-374 (PMC2194789; doi:10.1186/1471-2164-8-374)
Supplement: Additional file 1 — Distances between alternative TSSs within a sequence. [file 1471-2164-8-374-S1.doc]

**Additional file 1.** **Distances between alternative TSSs within a sequence.**

Next: between two neighboring ATSSs; Up: between the Most Upstream ATSS (MUTSS) to its following ATSS; Down: from the Most Downstream ATSS (MDTSS) to its preceding ATSS. U2D: from MUTSS to MDTSS.
