# Supplementary material for: MetaProm: a neural network based meta-predictor for alternative human promoter prediction
Source: BMC Genomics. 2007 Oct 17;8:374. doi: 10.1186/1471-2164-8-374 (PMC2194789; doi:10.1186/1471-2164-8-374)
Supplement: Additional file 2 — Distance between Transcription Start Site (TSS) and CpG island (annotated in UCSC). [file 1471-2164-8-374-S2.doc]

**Additional file 2. Distance between Transcription Start Site (TSS) and CpG island (annotated in UCSC).** The CpG islands are from annotation of UCSC hg17. ATSS: all promoters; MUTSS: most upstream promoter (most 5’ promoter); MTSS: middle promoter; MDTSS: most downstream promoters. Zero distance indicates the TSS is within the CpG island. The number in longer distance includes that in the short distance. MUTSS has 80% more chance to be in CpG island than MDTSS.
