# Supplementary material for: MetaProm: a neural network based meta-predictor for alternative human promoter prediction
Source: BMC Genomics. 2007 Oct 17;8:374. doi: 10.1186/1471-2164-8-374 (PMC2194789; doi:10.1186/1471-2164-8-374)
Supplement: Additional file 3 — Pairwise overlaps of correct predicted promoters between each PPP at high (50 bp) and low (2 kb) resolutions. [file 1471-2164-8-374-S3.doc]

**Additional file 3. Pairwise overlaps of correct predicted promoters between each PPP at high (50bp) and low (2kb) resolutions.**


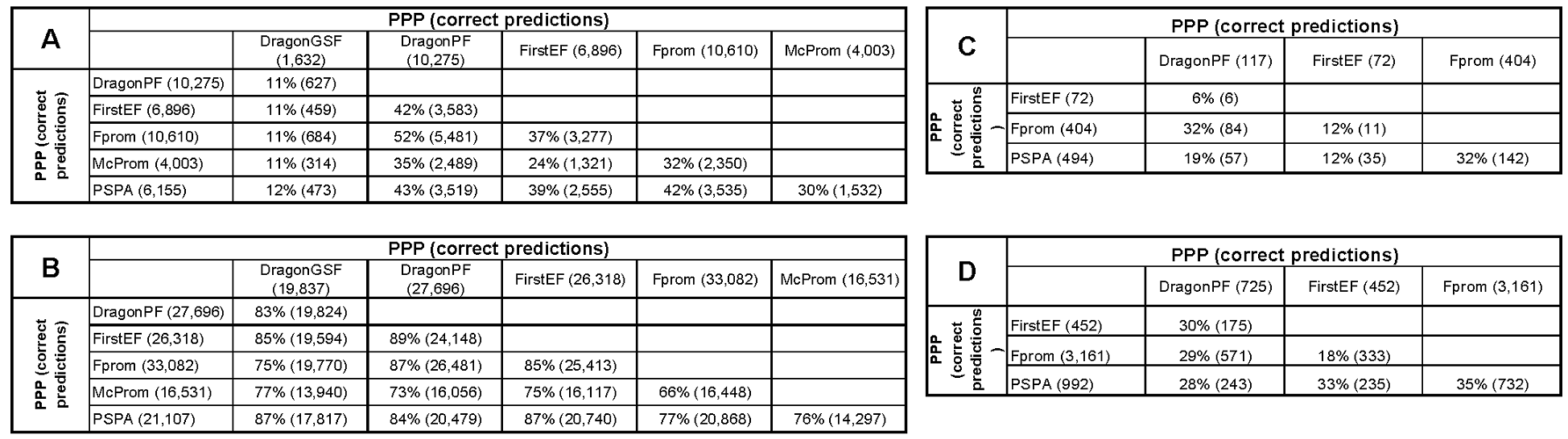


A, B for CpG rich promoters at high (50bp) and low (2kb) resolution respectively; C,D) on CpG poor promoters on high (50bp) and low (2kb) resolution respectively. The number in parenthesis is the count of correctly predicted promoters; the percentage number is calculated by the count of overlap divided by the mean of both PPPs.
