# Supplementary material for: MetaProm: a neural network based meta-predictor for alternative human promoter prediction
Source: BMC Genomics. 2007 Oct 17;8:374. doi: 10.1186/1471-2164-8-374 (PMC2194789; doi:10.1186/1471-2164-8-374)

**Additional file 4. Evaluation of MetaProm at high and low resolutions on genomewide promoter prediction.** A,B) on CpG rich promoters at high (50bp) and low (2kb) resolution respectively; C,D) on CpG poor promoters on high (50bp) and low (2kb) resolution respectively. The evaluations on metaProm are based on 10 fold cross-validation.


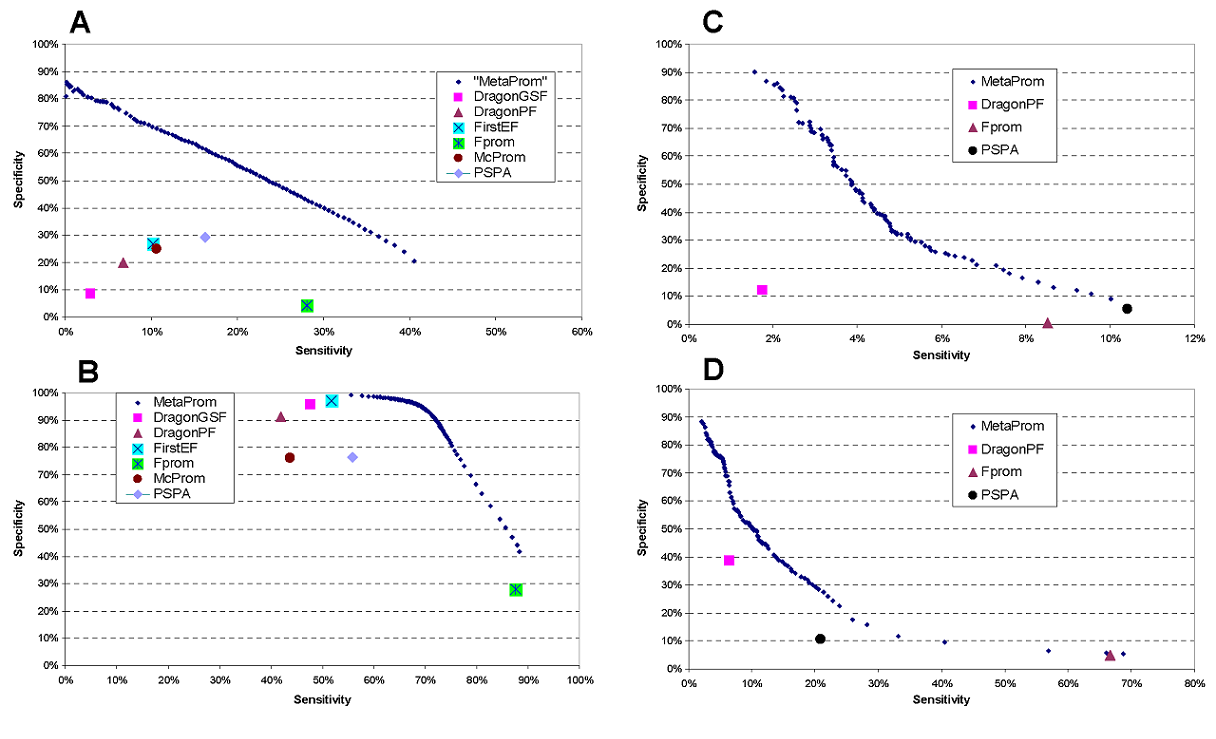

Supplement: Additional file 4 — Evaluation of MetaProm at high and low resolutions on genomewide promoter prediction. [file 1471-2164-8-374-S4.doc]
