# Supplementary material for: MetaProm: a neural network based meta-predictor for alternative human promoter prediction
Source: BMC Genomics. 2007 Oct 17;8:374. doi: 10.1186/1471-2164-8-374 (PMC2194789; doi:10.1186/1471-2164-8-374)
Supplement: Additional file 5 — Features used for ANN based MetaProm promoter prediction. [file 1471-2164-8-374-S5.doc]

Additional file 5. Features used for ANN based metaProm promoter prediction.

| **Category** | **Serial** | **Feature** | **Description** |
| --- | --- | --- | --- |
|  | 1 | id | identification of the sequence |
| **current prediction** | 2 | 0ppp | The PPP which produce this prediction |
|  | 3 | 0cpg | CpG-rich (1) or CpG-poor |
|  | 4 | 0score | PPP score or rank |
|  | 5 | l50 | number of other predictions in ±50bp |
|  | 6 | l100 | number of other predictions in ±100bp |
|  | 7 | l200 | number of other predictions in ±200bp |
|  | 8 | l500 | number of other predictions in ±500bp |
|  | 9 | l1k | number of other predictions in ±1kbp |
|  | 10 | l2k | number of other predictions in ±2kbp |
| **nearest prediction** | 11 | 1ppp | The PPP which produce this prediction |
|  | 12 | 1cpg | CpG-rich (1) or CpG-poor |
|  | 13 | 1dist | distance from the "current prediction" |
|  | 14 | 1dir | on upstream or downstream of "current prediction" |
|  | 15 | 1score | PPP score or rank |
| **second nearest prediction** | 16 | 2ppp | The PPP which produce this prediction |
|  | 17 | 2cpg | CpG-rich (1) or CpG-poor |
|  | 18 | 2dist | distance from the "current prediction" |
|  | 19 | 2dir | on upstream or downstream of "current prediction" |
|  | 20 | 2score | PPP score or rank |
| **CpG island** | 21 | cpg | CpG island type (is "current prediction" within, 100, 200, 500, 1k, or 2k bp away from CpG island, or no CpG island at all) |
|  | 22 | left | How far is the "current prediction" away from upstream of the CpG island |
|  | 23 | mid | How far is the "current prediction" away from middle of the CpG island |
|  | 24 | right | How far is the "current prediction" away from downstream of the CpG island |
|  | 25 | ct | How many CpG islands are there |
|  | 26 | len | length of the closest CpG island |
|  | 27 | gc | GC content of the CpG island |
|  | 28 | cg | CG ration of the CpG island |

We consider the features from current prediction (from a particular PPP), the closest two predictions by other programs, and CpG island information.
